# Dietary Cadmium Intake and Its Effects on Kidneys

**DOI:** 10.3390/toxics6010015

**Published:** 2018-03-10

**Authors:** Soisungwan Satarug

**Affiliations:** Centre for Kidney Disease Research and Translational Research Institute, The University of Queensland Diamantina Institute and Centre for Health Services Research, Woolloongabba, Brisbane 4102, Australia; sj.satarug@yahoo.com.au

**Keywords:** β2-microglobulin, body burden indicator, chronic kidney disease, dietary cadmium, exposure assessment, glomerular filtration rate, hypertension, *N*-acetyl-β-d-glucosaminidase, threshold limit, urine cadmium

## Abstract

Cadmium (Cd) is a food-chain contaminant that has high rates of soil-to-plant transference. This phenomenon makes dietary Cd intake unavoidable. Although long-term Cd intake impacts many organ systems, the kidney has long been considered to be a critical target of its toxicity. This review addresses how measurements of Cd intake levels and its effects on kidneys have traditionally been made. These measurements underpin the derivation of our current toxicity threshold limit and tolerable intake levels for Cd. The metal transporters that mediate absorption of Cd in the gastrointestinal tract are summarized together with glomerular filtration of Cd and its sequestration by the kidneys. The contribution of age differences, gender, and smoking status to Cd accumulation in lungs, liver, and kidneys are highlighted. The basis for use of urinary Cd excretion to reflect body burden is discussed together with the use of urinary *N*-acetyl-β-d-glucosaminidase (NAG) and β2-microglobulin (β2-MG) levels to quantify its toxicity. The associations of Cd with the development of chronic kidney disease and hypertension, reduced weight gain, and zinc reabsorption are highlighted. In addition, the review addresses how urinary Cd threshold levels have been derived from human population data and their utility as a warning sign of impending kidney malfunction.

## 1. Introduction

Cadmium (Cd) is a highly persistent environmental toxicant that exhibits higher rates of soil-to-plant transfer than other toxic heavy metals, such as lead (Pb) and mercury (Hg), making Cd a food-chain contaminant of great concern [1,2]. Further, Cd oxide (CdO), which is a highly bioavailable form of Cd, is present in cigarette smoke and polluted air, contributing to elevated Cd concentrations in blood, urine, and tissues of smokers, compared with non-smokers of similar age and gender [3,4]. Historically, consumption of rice contaminated with Cd from zinc mining discharge caused an outbreak of itai-itai disease that affected mostly women [5,6,7]. The hallmarks of itai-itai disease include severe kidney damage, generalized osteoporosis, osteomalacia, and multiple bone fractures [5,6,7]. 

To safeguard population health, safety limits of Cd in the environment and foodstuffs were established [8,9]. A safety limit of 3 mg/kg is applied to soils that are used for producing food crops for human consumption [9], while a 3 μg/L is applied to drinking water [8]. Safety limits, known as maximally permissible concentrations (MPC), have also been established for certain food crops and shellfish that are known as hyper-accumulators of Cd [9]. Currently, the MPC for potatoes is 0.1 mg/kg, while the MPC for rice is 0.4 mg/kg dry grain weight [9]. However, it is argued that MPC for rice should be reduced to 0.2 mg/kg dry grain weight to prevent adverse effects, especially in the populations that consume rice as a dominant energy (calorie) source [10]. This is typical of an Asian diet, which contributes to higher blood and urinary Cd levels in most Asian populations, when compared with other populations [4]. Asian subpopulations have been found to have the highest mean blood Cd among five ethnic groups studied in the U.S. National Health and Nutrition Examination Survey (NHANES), 2011–2012 [11].

In addition, a safe dietary Cd intake guideline and a urinary Cd threshold limit have been established by the Food and Agriculture Organization (FAO) and World Health Organization (WHO) Joint Expert Committee on Food Additives and Contaminants [12,13]. Currently, the FAO/WHO tolerable Cd intake level is 25 μg per kg body weight per month (0.83 μg/kg body weight/day or 58 μg/day for a 70-kg person), while a urinary Cd threshold level is 5.24 μg/g creatinine [14]. A threshold level is defined as a urinary Cd level at which 5% or 10% of the general population shows evidence of an adverse effect. The FAO/WHO tolerable intake level for Cd and the urinary Cd threshold limit were based on lifetime dietary Cd intake limit of 2000 mg per person, and critical kidney Cd concentration of 180–200 μg/g wet kidney weight [12,13]. 

It is increasingly apparent that adverse kidney effects occur at dietary Cd intake rates that are lower than the FAO/WHO estimated figures [4]. Urinary Cd levels below the threshold limit of 5.24 μg/g creatinine have also been associated with numerous adverse health effects, including chronic kidney disease (CKD) and type-2 diabetes, both of which are increasing in prevalence [4]. Further, cumulative lifetime Cd intake of 1300 mg, not 2000 mg, may increase the risk of developing itai-itai disease [10]. In light of these new data, the FAO/WHO-established safe intake guideline needs to be reassessed, as does the urinary Cd threshold limit. 

This review revisits aspects of dietary Cd intake and the effects on kidneys that underpin the FAO/WHO derivation of current threshold limit and tolerable intake levels for Cd. It highlights existing data on levels of Cd accumulation in human lungs, liver and kidneys that vary with age, gender, smoking status, and the presence of diseases. The basis for use of daily urinary Cd excretion rate to reflect total body content of Cd is discussed together with the biomarkers that have been used to quantitate kidney effects of Cd, notably *N*-acetyl-β-d-glucosaminidase (NAG) and low molecular weight proteins, such as β2-microglobulin (β2-MG). Data on urinary Cd threshold limits derived by the benchmark dose (BMD) method are provided along with their intended use as a warning sign of excessive Cd intake and adverse kidney effects.

## 2. Cadmium Sources and Intake Estimates 

Total diet study (TDS) and food frequency questionnaires (FFQ) have been used to estimate Cd intake rates in μg/day in an average consumer. The TDS is a food safety monitoring program, which is conducted by food authority agencies such as the U.S. Food and Drug Administration (FDA), the Food Standards of Australia and New Zealand (FSANZ), formerly known as the Australia and New Zealand Food Authority (ANZFA), and the European Food Safety Agency (EFSA). It is known also as the “market basket survey” because it involves collection of samples of foodstuffs from supermarkets and retail stores for quantitation of various food additives, pesticide residues, contaminants, and nutrients [14,15]. TDS provides a reasonable method to gauge the relative contribution of each food item to total intake of Cd. As expected, staples that are consumed in large quantities with high frequency contribute the most to total Cd intake. At present, TDS data are available for a limited number of countries, including the United States (U.S.), Australia, Sweden, France, Chile, Spain, Serbia, and Denmark, as reviewed in Satarug et al. [4]. Collectively, TDS data from these countries show that dietary Cd intake levels for the average consumer vary between 8 and 25 μg/day with staples (rice, potatoes, and wheat) forming 40–60% of total dietary Cd intake. Shellfish, crustaceans, mollusks, offal, and spinach are additional Cd sources [4]. 

In a U.S. study, FFQ estimated a mean dietary Cd intake of 10.4 μg/day (range: 1.74–31.6 μg/day) in women who participated in the Women’s Health Initiative [16,17,18]. In Spain, the mean for dietary intake derived from FFQ was 29.87 μg/day (range: 20.41–41.04 μg/day) for postmenopausal women and 25.29 μg/day (range: 18.62–35 μg/day) for premenopausal women [19,20]. In Japan, the mean Cd intake that was estimated by the FFQ was 26.4 μg/day in one study [21]. In another Japanese study, covering 30 locations nationwide, Cd intake ranged from 12.5 to 70.5 μg/day [22]. The majority of reported dietary Cd intake estimates are within the FAO/WHO tolerable level of 58μg/day for a 70-kg person, with an exception for certain locations in Japan, where intake exceeded the FAO/WHO safe intake guideline [22]. 

It is widely believed that the TDS method underestimates dietary Cd intake because the distribution of Cd in foods is highly skewed. This skepticism extends to most contaminants that reach foods through unpredictable processes. This problem is the likely cause of a failure to demonstrate an association between estimated Cd intake and the incidence of bone effects and breast cancer [17,19,20,23,24]. In striking contrast, urinary Cd excretion and blood Cd concentration correlate with the risk of developing of many diseases, even if the exposure to Cd is low [4]. A limited utility of TDS and FFQ data has led to an increased use of data from biomonitoring programs (Section 4). 

## 3. An Overview of Cadmium Kinetics

**Figure 1:**
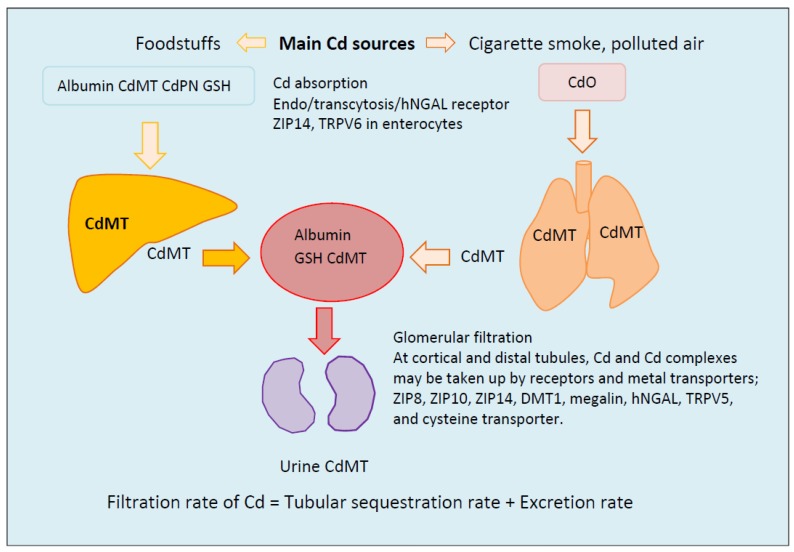
A schematic diagram showing cadmium uptake, transport and urinary excretion. Dietary Cd is absorbed and transported via the hepatic portal system to the liver, where it induces the synthesis of a specific metal binding protein, metallothionein (MT) to which Cd becomes tightly bound. MT-bound Cd is denoted as CdMT. Inhaled Cd induces MT in lungs, and CdMT is formed. CdMT formed by the enterocytes, liver and lungs enters the systemic circulation. Most cells, liver included, do not take up CdMT due to a lack protein internalization mechanism. In the kidneys, Cd, and Cd-complexes, including CdMT undergo glomerular filtration and either excretion or sequestration in proximal tubules. Because Cd in urine is bound to MT, excreted Cd is believed to have been filtered but not taken up by proximal tubules. Some urinary excretion of CdMT may result from re-entry of exosomes from proximal tubular cells into filtrate. CdMT = Metallothionein-bound Cd; CdO = Cadmium oxide; CdPN = Phytochelatin-bound MT; GSH = reduced glutathione; TRPV5 = Transient receptor potential vanilloid6TRPV5; TRPV6 = Transient receptor potential vanilloid6; hNGAL = human neutrophil gelatinase-associated lipocalin; ZIP = Zrt- and Irt-related protein of zinc transporter family; ZIP8 = Zrt- and Irt-related protein 8; ZIP10 = Zrt- and Irt-related protein10; ZIP14 = Zrt- and Irt-related protein 14.

Figure 1 provides an overview of Cd sources, uptake, transport, glomerular filtration, tubular sequestration, and excretion. Cd enters the body through the lungs and gastrointestinal tract in cigarette smoke, polluted air, and food. In cigarette smoke, Cd exists in oxide form (CdO), which is generated as tobacco burns. Cd in plant food crops is mostly in complex with phytochelatin.

Dietary Cd is taken up by the same transporter systems that the body uses to acquire calcium, iron, zinc, and manganese. These transporters may include divalent metal transporter1 (DMT1), Zrt- and Irt-related protein 14 (ZIP14, a member zinc transporter family), the Ca^2+^-selective channel transient receptor potential vanilloid6 (TRPV6), and human neutrophil gelatinase-associated lipocalin (hNGAL) receptor [25,26,27,28,29,30,31]. Cd bound to peptides, small proteins, and phytochelatin may be directly absorbed via transcytosis [30,31]. Cd of dietary origin is transported via the hepatic portal system to the liver, where it induces the synthesis of a metal binding protein, metallothionein (MT), which has a small mass (a molecular weight of 7 kDa) [32,33,34,35]. MT contains an unusually high molar content of cysteine indispensable for metal binding [33]. Cd becomes tightly bound to MT, and the complex is denoted as CdMT. Because Cd can exert toxicity as a free ion, CdMT is viewed as a detoxified form. Inhaled Cd induces MT in lungs, and CdMT is formed in situ. CdMT is released into the systemic circulation from enterocytes, liver, and lungs. Because liver cells do not take up the complex [32], CdMT from the gastrointestinal tract may be transported directly to kidneys [36].

In the kidneys, Cd in complexes with proteins, including MT, undergo glomerular filtration and may be taken up by the same receptors and transporter systems in cortical and distal tubules that are involved in reabsorption of proteins and nutrients. These may include ZIP8, ZIP10, ZIP14, DMT1, megalin, hNGAL receptor, TRPV5, and cysteine transporter. Previously, megalin and cubilin were suggested to mediate endocytosis of filtered CdMT [37,38], but this system exhibits only low affinity for CdMT. Thus the megalin and cubilin role in tubular CdMT uptake is questionable. To-date, the mechanisms for tubular CdMT internalization remain unresolved. 

Most excreted Cd is believed to have been filtered but not internalized by proximal tubules, because Cd in urine is bound to MT [39]. However, some urinary excretion of CdMT may result from re-entry of exosomes from proximal tubular cells into filtrate [32]. If this phenomenon is incorporated into another parameter, the rate of net tubular sequestration of Cd (TS_Cd_), then it follows that the filtration rate of Cd (F_Cd_) equals TS_Cd_ plus the excretion rate (E_Cd_).

The extremely long half-life of Cd in the human body [40,41] suggests that the majority of Cd that is taken from filtrate is retained indefinitely in tubular cells (a feature of cumulative toxicants). Because the majority of circulating Cd is thought to be bound to albumin, the typical ultrafilterable fraction of [Cd]_p_ ([Cd]_uf_); consequently, the difference between [Cd] _uf_ and E_Cd_ cannot be determined. Section 4 provides a further discussion on kinetics of Cd and interpretation of human urinary Cd excretion data.

### 3.1. Gastrointestinal Absorption of Cadmium

Animal and in vitro studies suggest that the absorption of Cd in the gastrointestinal tract is mediated by several transporter systems, which may include divalent metal transporter1, DMT1, Zrt- and Irt-related protein (ZIP) of zinc transporter family, namely ZIP14, and the Ca^2+^-selective channel, TRPV6 [25,26,27,28,29,30,31]. There is also evidence for absorption of dietary Cd by transcytosis mediated by the human neutrophil gelatinase-associated lipocalin (hNGAL) receptor [31]. The divalent metal transporter, DMT1 has the same high affinity for Cd as it does for iron (Km 0.5~1 μM) [25], and was thus thought to be the principal transporter for Cd in the enterocyte [15,16]. However, this transporter can only transport a free Cd ion, while Cd in food and intestinal environment is mostly bound to MT or phytochelatin. Nevertheless there are several potential Cd transporters in enterocytes. The zinc transporter, ZIP14, is highly expressed by the intestinal enterocytes [26,27], as is the Ca^2+^-selective channel, TRPV6 [28,29]. The calcium binding protein, calbindin may be involved in cytoplasmic transport of Cd, and further research is required to define the transport of Cd to the basolateral cell surface, where it exits the enterocyte into the circulation. 

Absorption rates for dietary Cd are influenced by the intake levels and body content of vital metals and elements. Women of reproductive age and children take up more Cd from diet than men because of their low body iron stores and iron deficiency. In a study of 448 healthy, non-smoking Norwegian women (aged 20–55 years, mean 38 years), those who had low body iron stores had 1.42-fold greater blood Cd (0.37 μg/L) than similarly aged women who had normal body iron stores [42]. In the same study, there was an inverse correlation between body iron stores and blood Cd and manganese and the prevalence of high levels of blood Cd and manganese was 26% in those with iron deficiency [42]. A Korean population study reported that women (aged 19–49 years) with iron deficiency had higher mean blood Cd level (1.53 μg/L) than those of the same age and normal body iron status (1.03 μg/L) [43]. Higher dietary zinc intake levels were associated with lower Cd body burden, as assessed by urinary Cd excretion levels [44].

### 3.2. Glomerular Filtration and Tubular Sequestration of Cadmium

Cd in the systemic circulation is concentrated in erythrocytes, and less than 10% is in the plasma, where it is associated with albumin, amino acids, and glutathione or tightly bound to MT [32]. 

Protein bound form of Cd is not readily taken up by most cells. Renal tubular cells are an exception because they are equipped for nutrient reabsorption, including virtually all of the proteins in filtrate [45]. In a study that used a microinjection technique, approximately 70–90% of Cd was taken up in the S1-segment of proximal tubules of the rat [46,47]. Uptake of Cd was reduced by a co-injection of zinc or iron [46]. Inhibition of Cd uptake by high concentrations of zinc, iron, and calcium has been demonstrated in another study, using perfused rabbit proximal tubules [47]. 

The zinc transporters ZIP8, ZIP10, and ZIP14 may mediate the tubular uptake of Cd [48,49,50]. Transgenic mice with three more copies of the ZIP8 gene accumulated twice as much Cd in the kidney following oral Cd exposure. Elevated ZIP8 expression at the apical membrane of proximal tubular cells accounted for their high sensitivity to Cd toxicity [48]. In mouse kidneys, ZIP8 and ZIP14 at the apical membrane are suggested to mediate the reabsorption of Cd and manganese, especially in the S3 segment of proximal tubules [49]. ZIP10 may also mediate tubular reabsorption of Cd since this zinc transporter is found in high abundance in renal cortical epithelial cells [50].

To-date, the molecular entities mediating the tubular uptake of CdMT have not been resolved (reviewed in [51,52]). Nevertheless, CdMT is taken up and degraded by endosomal and lysosomal protease enzyme systems in tubular cells with consequential release of toxic Cd ions into the cytoplasm. DMT1 was localized to the endosome and the lysosome in rat kidneys, and this suggested that DMT1 might mediate the release of toxic Cd ions [53,54]. This role for DMT1 was later confirmed in an experiment showing that the knockdown of DMT1 expression prevented CdMT-induced toxicity in the proximal tubular cell culture model [55]. 

The potential for DMT1 in the release of toxic Cd ions also suggests that kidney Cd toxicity may be magnified in iron deficiency state, the conditions in which DMT1 expression levels rise. The localization of FPN1 in the basolateral membrane of proximal tubular cells raises the possibility of involvement of FPN1in mediating Cd efflux. However, the high specificity of FPN1 for iron and cobalt not Cd [56], and only a small fraction of CdMT present at the basolateral membrane suggest that the majority of filtered Cd is retained in tubular cells. This retention may account for the long half-life of Cd in kidneys. The average half-life in kidneys is 14 years. It ranged from 9 to 28 years in a Japanese study [40] and was reported to be 45 years in a modeling study of Swedish kidney transplant donors [41]. The reasons for the large variation in Cd half-life are not apparent. 

### 3.3. Age-, Gender- and Organ-Differentiated Levels of Cadmium Accumulation 

In this section, data on measured levels of Cd in human organs are provided in Table 1, which includes data from two Japanese studies [57,58,59,60,61,62,63,64]. One was conducted on residents in an area without Cd contamination [62], and the other was conducted on patients with itai-itai disease and controls [63]. In Table 2 are data on Cd accumulation levels in men and women that include 36 cases of itai-itai disease, and there was only one male case of a total 36 cases [64]. This series exemplifies the preponderance of itai-itai disease in women. 

#### 3.3.1. Lower Kidney, Higher Liver Cadmium in Itai-Itai Disease Patients

Kidney Cd concentrations in itai-itai disease patients (aged 62–97 years) were dramatically lesser than controls (aged 46–87 years) (Table 1). Kidney Cd concentrations in these patients were 2 times lower than liver Cd levels; the mean of kidney cortex Cd was 36 µg/g wet weight, while the mean of liver Cd was 69.7 µg/g wet weight. The low kidney and high liver Cd in itai-itai disease patients provide strong evidence that diet was the dominant Cd source. Based on Cd content of rice grown in an area, where itai-itai disease was endemic, Cd intake levels were estimated to be over 200 µg/day or 1300 mg over lifetime [10]. The relatively small difference between cortical and medullary Cd content in elderly women with itai-itai disease provide also evidence for their nephron loss at these kidney Cd below a “critical” concentration, discussed below. This is because Cd is reabsorbed primarily by proximal tubules, and cortical Cd content would approach medullary Cd as proximal tubules are lost. 

Of note, current Cd risk assessment was based on critical kidney Cd concentration of 180–200 μg/g kidney cortex wet weight [13,14]. However, the mean kidney cortex Cd recorded for itai-itai disease patients, 36 µg/g wet weight (range: 8–133), was far below the critical concentration. This observation casts considerable doubt on the validity of these critical figures [65]. Because of nephron loss, Cd kidney concentrations in people dying from kidney disease were markedly lower than persons dying from other diseases [66].

As shown in Table 2, the mean liver Cd in Australian women was 1.74 fold higher than men [60]. Consistent with Australian data, the mean liver Cd in Japanese women in a low-Cd exposure group was 1.66 fold higher than men. Fractionally, the difference between men and women in kidney cortex content is smaller than the difference in hepatic content. A plausible interpretation is that women have lower iron stores, and adjustments to increase intestinal iron absorption lead to increased absorption and liver uptake of dietary Cd. Redistribution of hepatic Cd to the kidney may be sufficient to cause a higher kidney content of Cd as well, but not so great as to obscure the origin of the increased Cd burden. 

#### 3.3.2. Decline in Kidney Cadmium Content in Old Age

Excluding data from itai-itai disease patients, kidney Cd concentrations progressively increased with age, reaching a peak by 40–60 years. Of note, kidney Cd concentrations were consistently lower in the persons older than 60 years, compared to younger age groups. These data could be interpreted to suggest rising Cd exposure in recent times. Most likely, however, these data reflect age-related replacement of tubular cells by fibrosis, which is universal. The peak kidney cortex Cd level was 20, 22, 25, 42, 44, and 70 μg/g kidney cortex wet weight in Sweden, Greenland, Australia, Canada I, Canada II, and Japanese I study series, respectively. The kidney to liver Cd ratio in each corresponding kidney peak group was 25:1, 13:1, 17:1, 18:1, 20:1, and 37:1, respectively. This higher kidney Cd than liver is attributable to a continuing Cd influx (dietary, endogenous reservoirs notably liver, pancreas) to kidneys, as diagrammatically illustrated in Figure 1 and experimentally demonstrated [67,68]. In occupational exposure settings, inhaling relatively high-dose Cd in dust and fumes gave rise to high Cd levels in both liver and kidney (liver Cd 42.3 μg/g wet weight vs. kidney Cd 110 μg/g wet weight) in battery workers [69]. 

#### 3.3.3. Origin of Cadmium in Kidneys

In the Swedish study, a half of total kidney Cd content (10 μg/g kidney cortex) was estimated to come from food consumption, and the other half was attributed to cigarette smoking [57]. The majority of subjects with high kidney Cd levels (>50 μg/g) were women [57]. In Australian study [60], the mean kidney cortex Cd in high-lung Cd group was 6 μg/g ww higher than the medium-lung Cd group of similar age. The mean kidney Cd in smokers was 5 μg/g ww higher than non-smokers in a large British kidney only study [65]. Further, the mean kidney Cd was 9.7 μg/g ww higher in Australian women with high-lung Cd, when compared to men with similarly high-lung Cd levels although this value did not reach statistical significance. These findings may suggest high pulmonary absorption rates in women, and the redistribution of Cd from lungs to kidneys (Figure 1). 

In a study of living kidney transplant donors in Sweden, the rate of kidney Cd accumulation in non-smoker donors was 3.9 μg/g wet weight for every 10-year increase in age [70]. Smoking contributes to an additional 3.7 μg/g wet weight per 10 years. The rate of kidney Cd accumulation in Swedish non-smoker women with low iron stores (serum ferritin ≤ 20 μg/L) was 4.5 μg/g kidney wet weight for every 10-year increase in age.

#### 3.3.4. Urine, Blood and Kidney Cadmium: Data from Kidney Transplant Donors 

In an attempt to explore the utility of urine Cd to reflect cumulative lifetime exposure, Akerstrom et al. (2010) analyzed urinary Cd concentrations in relationship to the Cd levels in blood, and kidney biopsies of 109 living kidney transplant donors in Sweden (mean age 51 years, mean kidney Cd 12.9 μg/g wet weight) [71]. A urine-to-kidney Cd ratio of 1:60 was found to correspond to urinary Cd of 0.42 μg/g creatinine and kidney Cd content of 25 μg/g wet weight. In an equivalent analysis using Australian data, a lower urine-to-kidney Cd ratio of 1:20 was assumed because Australian subjects were 10 years younger than the Swedish subjects [72]. A urinary Cd of 1.25 μg/g creatinine corresponded to 25 μg/g Cd/g wet weight, a peak kidney Cd concentration [60]. Section 4 below provides a further discussion on the utility of urinary a quantitative measure of lifetime Cd exposure or intake. 

## 4. Does Urine Cadmium Reflect Total Body Content of Cadmium? 

Because TDS and FFQ data are of limited utility in health risk assessment of dietary Cd, there is a paradigm shift to use biomonitoring programs instead of dietary Cd intake estimates (Section 2). In most biomonitoring programs [73,74,75,76,77], single spot urine, and single blood samples are collected for quantitation of various toxicants, which often include ubiquitous toxic heavy metals, namely Cd, Pb, and Hg [73]. Other biologic specimens such as scalp hair and toe nails have sometimes been collected and analysed, but Cd levels in these specimens other than urine have not been rigorously evaluated. Their use remains questionable. Vacchi-Suzzi et al. (2016) have demonstrated good-to-excellent temporal stability of Cd in single spot or first morning void samples, thereby suggesting that urine Cd is suitable for use as a biomarker of long-term Cd exposure in epidemiologic research [78]. An adjustment of spot urine samples for urine creatinine excretion has also been addressed. Adding to the debate on use of urine Cd, this review highlights the fact that urinary Cd excretion can be best used to reflect total body content of Cd. Some certain circumstances that might partially invalidate the assumption for its use are also highlighted. 

The utility of urine Cd to reflect total body content of Cd is well founded by Cd levels that accumulated in human organs, notably livers and kidneys, such as those shown in Table 1 and Table 2. Kjellstrom and Nordberg (1978) developed the first Cd-toxicokinetics model, using Swedish autopsy data [57,79]. The kinetics model of Cd describes relationships among various parameters that govern the total body content of Cd. These include intake rate from oral and inhalational routes, absorption rate, systemic uptake rate, tissue distribution, half-life, and elimination through bile and urine. The original Kjellstrom and Nordberg model incorporated a single oral absorption rate of 5% for both men and women, and a half-life of Cd in kidneys as 20–30 years. It also assumed that Cd in kidneys comprises one-third of the total body content of Cd, and that 0.005% of the total body content of Cd is excreted in urine per day [79,80]. These assumptions underestimate body burden of Cd. Liver Cd content could be incorporated, given that combined liver and kidney Cd comprises two-thirds of the total body content of Cd. 

As more data and knowledge have become available, the Kjellstrom and Nordberg model parameters have now been modified [36,81,82,83,84,85]. The usefulness of modified models has been demonstrated [41,72,86,87,88]. For instance, modeling of the Cd concentrations in whole blood, plasma, urine, and kidney cortex samples from Swedish kidney transplant donors [41], a calculated daily systemic Cd uptake was 0.0052 μg Cd/kg body weight in men, and 0.0073 μg Cd/kg body weight in women. These systemic uptake rates correspond to an absorption rate between 1.7% and 2.5% in men and 2.4% and 3.5% in women (a 40% higher than men). In another modeling work [72], it is predicted that the dietary intake of Cd at current FAO/WHO tolerable monthly intake rate for 50 years will result in urinary excretion of Cd 0.70–1.85 μg/g creatinine in men and 0.95–3.07 μg/g creatinine in women. These urinary Cd levels have been associated with increased prevalence of CKD in the representative U.S. population (Section 5.3) and other diseases, including liver inflammation, osteoporosis, macular degeneration, hearing loss, depressive symptoms, obesity independent type 2 diabetes, cardiovascular disease, heart disease, breast cancer, and lung cancer in men, reviewed in Satarug et al. [4].

Apparently, urinary Cd excretion is a function of total body content of Cd, nephron numbers, tubular reabsorption capacity, the presence of other diseases, and other conditions, such as hypertension. Urinary Cd excretion rate can thus reflect accurately the total Cd body burden experienced by each individual person. However, interpretation of urinary Cd excretion rates should be done with caution, especially when used to assess Cd body burden in the elderly, people with diabetes, hypertension, and heavy smokers. Because of nephron loss, urinary Cd levels in these subjects can be expected to be lower than similarly age persons, who do not have these conditions. An effect of nephron loss on kidney Cd content is evident from a study that showed persons who died from kidney disease had lower kidney Cd levels than those who died from other diseases [66]. 

## 5. Measurement of Effects of Cadmium on Kidneys

Both FAO/WHO, the European Food Safety Agency (EFSA) used kidney tubular effects as the basis for derivation of safe intake levels and urinary Cd threshold limits [13,14,89,90]. Hence, kidney tubular impairment became the most widely studied effect of Cd. This effect is relevant, given that Cd uptake and accumulation in kidney tubular cells is the most extensive and the total amount of Cd in kidneys constitutes one-third of the total body burden (Section 3.3 and Section 4). Further, tubular cells contain large number of mitochondria that make them heavily reliant on autophagy to maintain homeostasis and highly susceptible to Cd-induced apoptosis [91,92,93]. In this section, conventional urinary biomarkers for the assessment of kidney tubular effects are discussed together with urinary Cd threshold levels for these effects. In addition, this section discusses chronic kidney disease (CKD) and other kidney-related effects of Cd that have recently emerged from human population studies. 

### 5.1. Biomarkers for Kidney Effects

A list of conventional urinary biomarkers that researchers have used to investigate tubular effects of Cd is provided in Table 3. These biomarkers are *N*-acetyl-β-d-glucosaminidase (NAG), lysozyme, total protein and albumin, β2-microglubin (β2-MG), α1-microglobulin (α1-MG), and kidney injury molecule-1 (KIM-1) [94,95,96,97,98,99,100,101,102,103,104,105,106]. Urinary levels of these biomarkers were adjusted to urinary creatinine excretion as most studies used single spot or void urine samples. Increased urinary excretion of nutrients, such as glucose, amino acids, calcium, and phosphorus has also been used to reflect tubular effect of Cd [107,108,109]. As indicated in Table 3, cut-off values of ≥100 mg/g creatinine were used for urinary total protein and ≥30 mg/g creatinine for urinary albumin [98]. These urinary total protein and albumin excretion levels are used also in CKD diagnosis [98]. Cut-off values for other markers, especially NAG, vary widely, depending on study populations and Cd exposure levels (see Section 5.2). Urinary NAG excretion is considered to be proportional to nephron numbers, as these enzymes mostly originate from tubular epithelial cells, and are released upon cell injury [99,107,108,109]. In a United Kingdom (U.K.) study, a dose–response relationship was observed between urinary Cd and NAG levels [110]. Further, urinary Cd of 0.5 μg/g creatinine was associated with 2.6- and 3.6-fold increases in the prevalence of urinary NAG >2 U/g creatinine, as compared with urinary Cd 0.3 and <0.5 μg/g creatinine, respectively [110]. 

Urinary α1-MG, β2-MG, and retinol binding protein (RBP) are all low-molecular-weight proteins that have traditionally been used to assess Cd tubular effects [6,99,100,101]. Another low-molecular-weight protein, namely cystatin C, has recently been evaluated in a rat model for suitability for use in Cd toxicity assessment [111]. Of note, data from Swedish kidney transplant donors suggested that urinary α1-MG excretion was a better marker than RBP or β2-MG, especially in persons with low urinary Cd excretion levels [105]. In the same Swedish donors study, a positive correlation was seen between kidney Cd concentrations and urinary α1-MG levels, while other biomarkers that were measured, such as KIM-1, RBP, and β2-MG did not correlate with kidney Cd concentrations [105]. The mean urinary α1-MG levels in study donors was 7.7 mg/g creatinine (range: 3.25–18.1), and the mean (range) kidney Cd concentrations was 15.0 μg/g wet weight (range: 1.45–55.4) [105].

By virtue of its small mass (MW ~12 kDa), β2-MG is filtered completely, as internalized by the proximal convoluted tubule through megalin-mediated endocytosis, and degraded [112,113,114]. Approximately 0.3% of filtered β2-MG is excreted in urine. Unlike NAG and lysozyme, β2-MG is produced by most cells in the body. Thus, elevated urinary β2-MG levels may reflect increased systemic β2-MG production or impaired tubular reabsorption [114]. In a sensitivity (renal toxicity) and specificity (non-renal organ toxicity) evaluation in rats, urinary β2-MG detected glomerular injury better than tubular damage [113]. Based on experimental data and clinical outcomes, it is suggested that urinary β2-MG is a predictor of glomerular filtration rate (GFR), and a high urinary β2-MG level could be interpreted to suggest primary glomerular pathologies, leading to protein load and competition of the filtered proteins (β2-MG included) for tubular reabsorption [114]. 

With respect to Cd effects, urinary β2-MG levels ≥1000 μg/g creatinine are considered to indicate severe and irreversible tubular impairment, while urinary β2-MG levels ≥300 μg/g creatinine are indicative of mild effects. In a prospective study in China, urinary β2-MG levels remained elevated (≥1000 μg/g creatinine) despite a reduction in urinary Cd levels from 11.6 to 9.0 μg/g creatinine over 8-year observation, while urinary albumin excretion recovered [115]. In line with Chinese study, a three-year follow-up study in Korea also suggested the irreversibility of severe tubular impairment in those who had urinary β2-MG levels exceeding 1000 μg/g creatinine [116]. 

### 5.2. Urinary Cd Threshold Levels

Currently, the benchmark dose (BMD) method has been used widely to derive a threshold or critical urinary Cd concentration to replace a formerly used no observed adverse effect level (NOAEL) or the lowest observe adverse effect level (LOAEL). Discussion on BMD method can be found in the reports by Crump (1984), Gaylor et al. (1998) and Gainsberg (2012) [117,118,119]. A threshold for tubular effects is defined as a urinary Cd level at which 5% or 10% of the population shows evidence of abnormal urinary excretion of tubular effect markers. All of the urinary Cd threshold levels shown herein considered a 10% level of risk above background [120,121,122,123,124,125]. Using data from 790 Swedish women, 53–64 years of age, urinary Cd 0.6–1.1 μg/g creatinine was derived as threshold levels for tubular toxicity [120]. The urinary Cd levels of 0.6–1.2 μg/g creatinine (0.8–1.6 μg/day) in men and 1.2–3.6 μg/g creatinine (0.5–4.7 μg/day) in women were found to be threshold for tubular toxicity, based on data from 828 Japanese subjects (410 men, 418 women), 40–59 years of age, who lived in areas without apparent pollution [121]. 

Based on data from 547 men to 723 women, aged 50 years or older who were residents of a high-Cd exposure area in Japan, urinary Cd levels of 2.1, 2.6 and 4.1 μg/g creatinine were derived as threshold for abnormal urinary excretion of protein, β2-MG, and NAG in men. The corresponding urinary Cd threshold levels in women were 1.5, 1.4, and 3.1 μg/g creatinine for protein, β2-MG, and NAG, respectively [122]. In this study of residents in a high-Cd exposure area, urinary Cd, β2-MG, and NAG levels were analysed as continuous variables, not being categorized by cut-off values [122]. In a Chinese study, urinary Cd of 0.57–1.84 μg/g creatinine was identified as threshold levels for abnormal urinary β2-MG levels (≥1065 μg/g creatinine), while urinary Cd of 1.19–1.37 μg/g creatinine was identified as threshold levels for abnormal urinary NAG levels (≥5.67 units/g creatinine) [123]. A study of 6103 residents in five high-Cd exposure areas in China, urinary Cd threshold level for a permanent tubular effect (urine β2-MG levels ≥ 1000 μg/g creatinine) in men was 2 μg/g creatinine, and 1.69 μg/g creatinine in women [124]. 

Based on data from occupationally exposed populations in China, urinary Cd threshold levels for abnormal urinary excretion of NAG, β2-MG, and albumin were 2.7, 3.4, and 4.2 μg/g creatinine, respectively [125]. The cut-off values used were 9.8 units/g creatinine, 187.6 μg/g creatinine, and 13.5 mg/g creatinine for NAG, β2-MG, and albumin levels, respectively. These urinary Cd threshold levels in occupationally exposed subjects were slightly higher than in environmentally exposed populations, but all were lower than the FAO/WHO figure. In the same study, a urinary Cd threshold level for abnormally high urinary MT levels (≥388.8 ng/g creatinine) was 3.1 μg/g creatinine [125]. 

None of the urinary Cd threshold levels that were derived from environmental and occupational exposure situations exceed the FAO/WHO established threshold of ≥5.24 μg/g creatinine. Thus, the FAO/WHO figures do not offer health protection. Although Cd has been increasingly associated with disease in tissues and organs other than kidneys [3,4], urinary threshold levels have been derived mostly based on tubular effects. A wide diversity of Cd toxicity levels and toxicity targets requires that urinary Cd threshold levels should be derived for the adverse effects of Cd in many other tissues, such as bone, liver, and retina. In this way, the tissue/organ most sensitive to Cd can be identified, and this organ should be considered as a critical target of Cd toxicity for the derivation of an evidence-based threshold to provide sufficient protection. 

### 5.3. Cadmium and Urine β2-MG: A Revisit

Elevated urinary β2-MG levels that have often been found in people with increased Cd body burden have long been dismissed and have been deemed to not be of clinical relevance. It has further been argued that associations between urinary Cd and commonly measured urinary biomarkers, notably albumin and β2-MG do not reflect toxicity, but reverse causality [126]. In theory, albumin in urine could interfere competitively with CdMT for tubular reabsorption, and thereby increase Cd excretion. Albumin could also impede β2-MG reabsorption. If the patient has a renal disease that is not related to Cd that is rapidly reducing GFR, then Cd excretion would be increased. 

However, experimental and clinical outcome data suggest that high urine β2-MG levels could be a result of glomerular pathologies, causing protein load and competition of the filtered proteins for tubular reabsorption [112,113,114]. Supporting a potential connection between elevated Cd body burden and GFR reduction is an association between higher blood Cd levels and lower eGFR values in adult participants in NHANES 2007–2012 [127]. In addition, a Korean population study has shown that blood Cd levels in the highest tertile were associated with 1.85 mL/min/1.73 m^2^ (95% CI: −3.55, −0.16) lower eGFR values, when compared with the lowest tertile [128]. A population-based prospective study in Japan reported that there was a 79% increase in risk of having accelerated GFR decline (10 mL/min/1.73 m^2^ over five-year observation period) in those who had urinary β2-MG levels ≥ 300 μg/g creatinine [102]. In a cross-sectional study, urinary β2-MG levels ≥ 145 μg/g creatinine were associated with an increased risk of developing hypertension, as compared with urinary β2-MG levels ≤ 84.5 μg/g creatinine [103]. 

### 5.4. Cadmium and Chronic Kidney Disease

Chronic kidney disease (CKD) is a cause of morbidity and mortality, and its prevalence is rising worldwide [129,130]. CKD is defined as an estimated glomerular filtration rate (eGFR) that is below 60 mL/min/1.73 m^2^ or urinary albumin to creatinine ratio above 30 mg/g [129,130]. CKD is more prevalent in people with hypertension; the CKD prevalence in 17,794 participants (aged ≥ 20 years) in the U.S. NHANES 1999–2006 was 13.4%, 17.5%, 22%, and 27.5% in those with normal blood pressure, prehypertension, undiagnosed hypertension, and diagnosed hypertension, respectively [131]. CKD prevalence rate in normotensive participants of 13.4% exceeds the 5% acceptable disease prevalence in the general population. In this NHANES 1999–2006 data, urinary Cd levels > 1 μg/L were associated with 41–63% increases in the prevalence odds of CKD and albuminuria [132]. In a separate analysis, blood Cd level of 0.6μg/L or higher showed also an association with risks of developing CKD and albuminuria in NHANES 1999-2006 adult participants [133]. 

In a recent NHANES 2007–2012 cycle, the overall mean urine Cd level of 0.35 μg/L and mean blood Cd of 0.51 μg/L were lower, when compared with the NHANES 1999–2006 [128]. Such reduction in body burden of Cd in the U.S. population was attributable to a reduction in smoking prevalence, but there was no evidence for a reduction in Cd intake from dietary sources. Despite a reduced population mean urine and blood Cd levels, blood Cd levels > 0.53 μg/L were associated with two-fold increases in prevalence of low GFR (OR 2.21, 95% CI 1.09–4.50) and albuminuria (OR 2.04, 95% CI 1.13–3.69) in an analysis included a subset population (the NHANES 2011–2012) [134]. This was close to the blood Cd level of 0.6 μg/L that was found to be associated with increased risks of developing CKD and albuminuria in adult participants in the NHANES 1999–2006 [133]. 

Further, an additional increase in risk of albuminuria was seen in Cd-exposed subjects with low zinc status (low serum zinc levels) as OR rose to 3.38 (95% CI, 1.39, 8.28), comparing with those who had higher zinc status [134]. This raises the possibility that CKD results from an increased body burden of Cd. These two conditions have been associated in two NHANES cycles and in cross-sectional studies of other populations, including Korea and China [135,136]. It is also possible that increased urinary Cd, which is the accepted indicator of body burden, may be a consequence of albuminuria or CKD, rather than the cause. Albuminuria may cause also an increase loss of zinc through urine, resulting in trace metal deficiency. Evidence for increased urinary zinc excretion in Cd-exposed subjects in the absence of albuminuria would suggest that Cd can induce urinary loss of zinc whether albumin is present in filtrate or not. 

GFR falls if a disease causing albuminuria also destroys glomeruli, or if toxic substances destroy tubular cells after reabsorption from filtrate. Blood pressure rises if GFR falls for any reason, and GFR may fall as a consequence of damage due to hypertension. At a given rate of influx of Cd into plasma from all sources, the plasma Cd concentration is likely to rise as GFR falls. Blood Cd levels ≥ 0.4 μg/L were associated with increased risk of hypertension in Caucasian women (OR 1.54, 95% CI 1.08–2.19), and in Mexican–American women (OR 2.38, 95% CI 1.28–4.40) who participated in the NHANES 1999–2006 [137]. Association between elevated Cd body burden and hypertension development, especially in women, was also seen in Koran and Canadian population studies [138,139]. This would be expected as women are at risk of Cd toxicity due to enhanced Cd uptake (Section 3.1). 

Hypertension in Thai women, who were environmentally exposed to Cd, has been associated with increased urinary levels of 20-hydroxyeicosatetraenoic acid (20-HETE), which plays an indispensable role in renal salt balance and blood pressure control [140]. Urinary 20-HETE levels above the median 469 pg/mL were associated with a 90% increase in prevalence odds of hypertension, a four-time increase in odds of having higher urinary Cd levels, and a 53% increase in odds of having higher urinary β2-MG levels [140]. These results link urinary 20-HETE levels to blood pressure increases in Cd-exposed women, thereby providing a plausible mechanism for associated hypertension development.

### 5.5. Cadmium and Reduced Weight Gain 

The body content of Cd assessed by urinary and/or blood Cd levels showed an inverse association with body mass index (BMI), central obesity, and risks of weight gain, and obesity in both children and adults. These have consistently been observed across populations, including the U.S., Belgium, Canada, Korea, and China [77,141,142,143,144,145,146]. In the U.S. NHANES 1999–2002 participants, an inverse association between body burden (urinary Cd levels) and central obesity was noted [141], while an inverse association between blood Cd and BMI was seen in the NHANES 2003–2010 participants [142]. The Canadian Health Survey 2007–2011 has reported that non-smokers with higher BMI had lower blood and body content of Cd, as reflected by urinary Cd excretion [77]. In a Chinese study, urinary Cd levels that were equivalent to or greater than 2.95 μg/g creatinine were associated with a reduced risk of being overweight [146]. In a Korean study, higher blood Cd levels that were associated with BMI in Korean men (40–70 years) with mean blood Cd of 1.7 μg/L, and mean urinary Cd of 2.13 μg/g creatinine [145]. 

These observation of lower BMI and lower risk of overweight with higher levels of total body content of Cd are consistent with a reduction in body weight after renal glucose reabsorption is reduced by therapeutically administered sodium glucose cotransporter 2 (SGLT2) inhibitors [147,148]. This was an unexpected outcome from a therapeutic application of glucose reabsorption inhibitors for management of hyperglycaemia or as anti-diabetic drugs [147,148]. This new class of anti-diabetic drugs also show promise in weight reduction and blood pressure control [148,149]. A potential effect of Cd on glucose reabsorption and its contribution to altered body weight homeostasis are discussed below. 

Glucose reabsorption in kidneys is mediated by SGLT2, localized to cortical proximal tubular cells, where the bulk of calorie as glucose (an approximate of 160 to 180 gm) is reabsorbed and returned to the systemic circulation daily, under normal physiological conditions [147,148]. An effect of Cd on the activity and/or abundance to SGLT2 in the proximal tubules was deduced from an observation of glycosuria in the subjects with high Cd body burden without hyperglycaemia [94,108]. In a Swedish study, subjects with higher levels of Cd body burden were found to excrete higher levels of citrate, 3-hydroxyisovalerate, and 4-deoxy-erythronic acid, which are the biomarkers of mitochondria [150]. In this study, a positive association was seen also between urinary Cd and 8-oxo-deoxyguanosine, a marker of increased systemic oxidative stress. Increased urinary citrate levels may be secondary to Cd effect on tubular reabsorption of filtered citrate rather than the spill out of citrate and other organic anions due to mitochondrial damage. This is because no correlation was seen between urinary citrate and NAG levels. 

Interestingly, a study of 168 Thai subjects in a high-Cd exposure area and 100 controls also observed increases in urinary citrate levels with Cd, and the authors suggested that Cd may have a direct effect on mitochondrial citrate metabolism because of the strength of an association between urinary Cd and urinary citrate levels persisted after adjustment for age, smoking status, and severity of tubular impairment, assessed by urine β2-MG levels [151]. Support of these Swedish and Thai data included an effect of Cd on mitochondrial oxidative phosphorylation in tubular cells, causing a reduction in ATP output [91], and a fall in the abundance of the Na/K-ATPase and its sodium transport activity in tubular epithelial cells that were treated with Cd [52,152] 

### 5.6. Cadmium and Depressed Serum Zinc: Role for Impaired Zinc Reabsorption

Zinc is the second most abundant metal in the body, and the contribution of kidneys to zinc homeostasis is well established [153]. However, there are limited studies that assessed Cd effects on zinc homeostasis and tubular zinc reabsorption. A potential Cd effect on zinc homeostasis comes from the Belgian study, including 959 men and 1018 women, aged 20–80 years, which observed depressed serum zinc levels in those with elevated Cd body burden [154]. The depressed serum zinc levels persisted even after subjects with occupational exposure to metals were excluded [154]. Likewise, reduced serum zinc levels were found to be associated with higher blood Cd levels in another study of 299 healthy Croatian men, 20–55 years of age [155]. Of note, women in the Belgian study were found to have lower serum zinc (mean 12.6 μM, range 6.3–23.2 μM) than men (mean 13.1 μM, range 6.5–23.0 μM). A Thai study observed also lower mean serum zinc in women (18.4 μM), as compared to men (21 μM). In this Thai study, lower fractional zinc reabsorption levels were associated with higher Cd body burden, higher serum copper to zinc ratios, and higher tubular impairment levels, as assessed by the urinary excretion of β2-MG [156].

In an Australian autopsy study, levels of zinc in liver and kidney cortex decrease with rising age and Cd levels [157]. In this study, liver and kidney MT levels were not quantified. However, a regression model analysis showed that a large fraction of zinc in kidney cortex was associated with the MT pool [157]. In the liver, however, there was much less zinc in MT than in non-MT pool. These findings are consistent with MT metamorphism, which explains the different zinc and copper contents in MT molecules from different tissues and organs [33]. Australian data suggested that zinc in kidney was mostly bound to MT, whereas the majority of zinc in liver was not associated with MT. 

By immunohistochemistry, in human kidneys cortex MT-1/2 was found mostly in the cytoplasm and nuclei of proximal tubular cells, and to a less extent in distal tubules, but not in the glomeruli, or associated interstitial and vascular elements [158]. MT-1/2 was found in proximal and distal tubules of rat kidneys [152]. Another form of MT (MT-3) was found to be expressed in high abundance in human kidneys, especially in the distal tubule [159]. However, MT-3 is known to bind Cd less vividly than MT-1/2. In primary culture of human proximal tubular cells, MT-1/2 was induced by as little as 0.5 μM Cd [158]. A study in Thai subjects in high-Cd exposure area reported that MT transcript levels in leucocytes increased with increasing blood and urinary Cd levels [160]. Further, the high levels of MT transcript in leucocytes were associated with reduced urinary albumin and β2-MG levels, suggesting a reduction in Cd toxicity as MT levels increased [160]. 

The influence of MT and Cd concentrations on kidney zinc and copper concentrations is suggested by data from two additional Turkish population studies [161,162]. In one study, the AG and GG variants of the MT2A gene promoter were associated with higher kidney cortex Cd levels than the AA variant, but there were no differences in zinc or copper levels [161]. In the other study, the GG variant was associated with higher blood Cd levels, but lower blood Zn levels, when compared with the AA and AG variants [162]. In contrast, data from a large Japanese (Nagoya) study (749 men and 2025 women, aged 39–75 years) observed no differences in serum MT, Cd, or zinc levels across the three MT2A promoter variants (AA, GA, GG). Of interest, the GG variant was associated with an increased risk of developing CKD or diabetes in Japanese subjects [163]. Collectively, these findings suggest a closer link between Cd, MT, and zinc homeostasis than copper and further investigation is required to dissect the link that may exist between MT, Cd, zinc, and the development of CKD and diabetes. 

## 6. Conclusions

Currently, dietary Cd intake is estimated to be between 8 and 25 μg/day in various populations. These are within the FAO/WHO tolerable intake level of 58 μg/day for a 70-kg person. Kidney cortical Cd concentrations increase progressively with age, reaching a peak by 40–60 years. The recorded peak kidney cortical Cd accumulation of 20–70 μg/g wet weight is also well below critical kidney Cd concentrations of 180–200 μg/g kidney. However, population research data reviewed herein suggest that Cd has adverse effects on kidneys at Cd intake rates and kidney Cd concentrations that are lower than these estimated figures. Elevated urinary excretion of a low-molecular weight protein β2-MG and NAG, termed tubular proteinuria and enzymenuria, have been used to reflect kidney toxic effects of Cd ever since the discovery of β2-MG in urine of Cd-exposed humans. Supporting an effect of Cd on kidneys is an association between GFR reduction and increased urinary β2-MG levels. 

Other possible kidney effects of Cd may include an inhibition of glucose reabsorption, and reduced zinc reabsorption by the kidneys, thereby affecting energy and zinc homeostasis. These adverse effects of Cd on the kidneys have been observed at urinary Cd levels below an established threshold limit of urinary Cd of 5.24 μg/g creatinine. These observations cast considerable doubt on the validity of current “tolerable” intake level for Cd and its “critical” kidney concentrations. There is an urgent need to reassess the Cd toxicity threshold limit, as it currently does not afford the protection that it should to prevent excessive Cd exposure and its adverse effects. Public health measures are needed to minimize Cd contamination of the food-chain. Risk reduction measures are also required to reduce air pollution, smoking, workplace exposure, and gastrointestinal absorption of Cd, especially for populations that are deemed to be of increased risk of exposure from all sources.

## Figures and Tables

**Table 1:** Age- and organ-differentiated levels of cadmium accumulation.

Country	Age/Organs	Cadmium (μg/g Wet Tissue Weight)								
Sweden [57]	Age	0–9	10–29	30–39	40–59	60–79	80–99			
	Liver	0.7	0.6	0.6	0.8	1.0	0.6			
	Kidney	2.4	8.8	18.0	19.9	15.0	7.1			
	K/L ratios	3.4:1	15:1	30:1	25:1	15:1	11:1			
Canada I [58]	Age		1–20	21–40	41–60	61–80	81–90			
	Liver		1.0	1.7	2.3	2.2	0.7			
	Kidney		5.4	26.3	41.8	16.4	6.8			
	K/L ratios		5.4:1	16:1	18:1	7.5:1	9.7:1			
Canada II [59]	Age	<10	10–19	20–29	30–39	40–49	50–59	60–69	70–79	>79
	Liver	0.3	0.7	1.4	1.5	1.6	2.2	1.8	1.5	2.5
	Kidney	4.5	5.2	6.8	18.9	41.2	44.2	32.7	23.6	22.8
	K/L ratios	15:1	7.4:1	4.9:1	13:1	26:1	20:1	18:1	16:1	9:1
Australia [60]	Age	2–7	10–19	20–29	30–39	40–49	50–59	60–69	70–79	80–89
	Lung	0.01	0.04	0.22	0.11	0.30	0.14	0.12	0.08	0.03
	Liver	0.21	0.71	0.65	0.95	1.45	0.93	0.94	2.14	1.0
	Kidney	1.63	5.44	9.80	17.8	25.0	22.1	21.6	31.7	8.6
	K/L ratios	7.8:1	7.7:1	15:1	19:1	17:1	24:1	23:1	15:1	8.6:1
Greensland [61]	Age			19–29	30–39	40–49	50–59	60–69	70–79	80–89
	Liver			1.4	2.0	1.7	0.8	1.6	2.6	1.6
	Kidney			12.3	17.8	22.3	18.3	15.8	15.4	5.2
	K/L ratios			8.8:1	8.9:1	13:1	23:1	9.9:1	5.9:1	3.3:1
Japan I [62]	Age	0–1	2–20	21–40	41–60	61–95				
	Liver	0.05	1.1	2.3	1.9	3.6				
	Kidney	0.61	8.4	33.3	69.8	52.3				
	K/L ratios	12:1	7.6:1	15:1	37:1	15:1				
Japan II [63] ^a^	Age				46–87	62–97				
	Liver				11.9	69.7				
	Cortex				87.3	36.0				
	Medulla				39.1	25.3				
	K/L ratios				7.3:1	0.5:1				

K/L = Kidney cortex to liver Cd ratio; ^a^ = Data are from itai-itai disease patients (aged 62–97 years) and controls (aged 46–87 years) [63].

**Table 2:** Gender differences in levels of cadmium accumulation.

Country	Age/Organs	Cadmium Concentration (μg/g Wet Weight)					
		Males			Females		
		N	Mean	Range	N	Mean	Range
Australia [60]	Age (years)	43	37.05	2–89	18	42.11	3–86
	Lung	43	0.11	0.001–1.15	18	0.17	0.001–1.45
	Liver	43	0.78	0.10–3.23	18	1.36	0.18–3.95
	Kidney	43	14.6	0.72–43.03	18	18.1	1.67–63.25
Japan III [64]	Itai-itai disease diagnosis						
	Age (years)	1	94	-	35	78.5	61–90
	Liver	1	139.0	-	35	62.4	14.4–170.2
	Kidney cortex	1	58.3	-	33	25.6	9.7–112.5
	Kidney medulla	1	36.6	-	32	20.8	8.9–66.7
	Pancreas	1	92.0	-	23	42.8	11.1–102.8
	Thyroid	1	132.1	-	22	35.0	1.9–171.0
	Heart	1	2.9	-	25	0.8	0.2–4.8
	Muscle	1	16.1	-	25	8.5	3.5–14.6
	Aorta	1	3.9	-	24	2.5	0.3–4.7
	Bone	1	2.5	-	25	1.6	0.2–3.8
Japan III [64]	Residents of a non-polluted area						
	Age (years)	36	71.4	60–85	36	72.7	60–91
	Liver	36	7.9	1.3–33.3	36	13.1	3.1–106.4
	Kidney cortex	36	72.1	19.4–200	35	83.9	3.9–252.9
	Kidney medulla	36	18.3	3.5–76.4	35	24.5	4.0–105.0
	Pancreas	7	7.4	3.0–25.9	16	10.5	2.5–29.8
	Thyroid	5	10.6	3.8–35	16	11.9	3.9–56.4
	Heart	7	0.3	0.1–0.5	17	0.4	0.1–1.3
	Muscle	7	1.2	0.3–3.2	16	2.2	0.8–12.4
	Aorta	5	1.0	0.4–2.5	16	1.1	0.3–3.0
	Bone	5	0.4	0.2–0.6	16	0.6	0.2–1.6

**Table 3:** Urinary biomarkers for assessment of kidney effects of cadmium.

Biomarkers	Abnormal Values	Interpretations
NAG	>4 U/g creatinine	Tubular injury, mortality [94,95,96].
Lysozyme	>4 mg/g creatinine	Tubular injury [97].
Total protein	>100 mg/g creatinine	Glomerular dysfunction, CKD [98].
Albumin	>30 mg/g creatinine	Glomerular dysfunction, CKD [98].
β2-MG	≥1000 μg/g creatinine	Irreversible tubular dysfunction [6,99,100,101].
β2-MG	≥300 μg/g creatinine	Mild tubular dysfunction, rapid GFR decline [102].
β2-MG	≥145 μg/g creatinine	Increased risk of hypertension, compared with urinary β2MG levels ≤84.5 μg/g creatinine [103].
α1-MG	≥400 μg/g creatinine	Mild tubular dysfunction [104,105]
α1-MG	≥1500 μg/g creatinine	Irreversible tubular dysfunction [6,104].
KIM-1	≥1.6 mg/g creatinine in men, ≥2.4 mg/g creatinine in women	Kidney injury, urinary KIM-1 levels correlate with blood Cd levels [106].

NAG = *N*-acetyl-β-d-glucosaminidase; β2-MG = β2-microglobulin; α1-MG = α1-microglobulin; KIM-1 = Kidney injury molecule-1.
